# Phase-specific trunk kinematics during functional mobility in very old adults

**DOI:** 10.3389/fpsyg.2026.1805004

**Published:** 2026-10-27

**Authors:** Chao-Fu Chen, Jie Meng, Lin Zhu, Xuqiang Sun

**Affiliations:** 1 School of Physical Education and Sport, Henan University, Kaifeng, China; 2 School of Rehabilitation, Henan Medical University, Xinxiang, Henan, China; 3 Physical Education College, Zhengzhou University of Industrial Technology, Zhengzhou, China; 4 School of Physical Education and Sport, Xinxiang Institute of Engineering, Xinxiang, China

**Keywords:** functional mobility, older adults, phase-specific analysis, postural control, trunk kinematics

## Abstract

**Introduction:**

Age-related declines in mobility and postural control substantially increase fall risk among older adults. Functional mobility is commonly evaluated using overall task completion time, yet this aggregated measure may obscure phase-specific movement deficits and underlying kinematic control mechanisms. This study aimed to examine phase-specific temporal characteristics and trunk kinematic patterns during a continuous functional mobility task in a sample of very old adults (mean age, 87.95 years).

**Methods:**

Twenty-one older adults (87.95 ± 2.60 years) performed a timed up and go–based task consisting of standing up, walking, turning, returning, and sitting down. Trunk kinematics were recorded using a wearable inertial measurement unit to capture tri-axial accelerations and trunk orientation angles in the sagittal, frontal, and transverse planes. The task was segmented into six phases. Temporal parameters and trunk kinematic variables were analyzed using Pearson correlation analysis.

**Results:**

Overall task completion time was strongly correlated with the standing-up phase (*r* = 0.743, *p* < 0.01) and the walking-after-turn phase (*r* = 0.782, *p* < 0.01). During standing up, vertical trunk acceleration was positively associated with mediolateral acceleration (*r* = 0.561, *p* < 0.01). Trunk orientation measures were positively correlated across planes during standing up, whereas sagittal and transverse plane angles were highly correlated during sitting down (*r* = 0.951, *p* < 0.01).

**Conclusion:**

In this small sample with a mean age of approximately 88 years, phase-specific durations and trunk kinematic measures showed descriptive patterns of association. The results are hypothesis-generating and do not establish causal mechanisms, fall prediction, or superiority over total TUG time. Replication with repeated measurements and a larger, well-characterized sample is needed.

## 1 Introduction

With rapid global population aging, age-related declines in mobility and the associated increased risk of falls among older adults have become major concerns for public health and long-term care systems (Xu et al., 2022; Khow and Visvanathan, 2017). Sequential transitional movements, such as rising from a chair, walking, turning, and sitting down, are frequently performed in daily life and are also movement situations in which falls commonly occur among older adults (Tiago Horta, 2024). Impaired performance in these functional tasks has been associated with reduced lower-limb muscle strength, diminished postural stability, and limitations in multisegmental coordination (Gianfredi et al., 2025). Therefore, objective and mechanistically meaningful quantitative analyses of daily functional mobility tasks are important for understanding age-related mobility deterioration and for developing targeted preventive intervention strategies (Jaul and Barron, 2017).

In clinical and community settings, functional mobility is commonly assessed using overall task completion time as the primary outcome measure. The Timed Up and Go test is widely used because it is simple, feasible, and clinically practical for evaluating functional performance in older adults (Savva et al., 2013). However, total completion time represents a single aggregated outcome and may not fully capture the movement characteristics of individual task phases, such as sit-to-stand, walking, turning, return walking, and stand-to-sit. Given the heterogeneity of age-related functional decline, older adults may present different limitations during specific phases of the task. Therefore, relying solely on total task duration may obscure phase-specific temporal deficits and underlying movement-control characteristics that are relevant to functional mobility and fall risk (Bloom et al., 2015).

Recent advances in wearable sensing technologies have enabled the measurement of acceleration and body orientation in naturalistic environments, providing new opportunities to investigate dynamic stability and postural control in older adults (Perez and Zeadally, 2021; Lou et al., 2018). Beyond lower-limb or gait-related parameters, the trunk plays a pivotal role in maintaining whole-body stability and coordinating multisegmental movements during functional mobility, particularly during postural transitions such as standing up and sitting down (Wong et al., 2015). However, existing studies on functional mobility in older adults have largely focused on overall performance measures or isolated task phases, while systematic segmentation and comparative analyses of sequential phases—such as rising, walking, turning, return walking, and sitting down—remain limited (Dolson et al., 2022; Shajari et al., 2023).

Within this context, instrumented Timed Up and Go tests have increasingly been used to provide more detailed quantitative information beyond total completion time alone (Ortega-Bastidas et al., 2023). Compared with traditional time-based TUG assessment, instrumented approaches can support the analysis of temporal, acceleration, angular, and movement-related parameters during different components of the task. Previous studies have also shown that TUG-related measures are useful for describing functional mobility characteristics and screening fall-related risk in older adults (Kear et al., 2017; Chow et al., 2019). Nevertheless, the interrelationships between trunk accelerations and multiaxial trunk orientation angles, including flexion–extension, lateral bending, and axial rotation, have not yet been comprehensively examined across different phases of a continuous TUG task.

The present study examined phase-specific durations and trunk kinematics during a continuous 3-m TUG-based task in very old adults. The aims were to examine associations between total and phase durations, describe tri-axial trunk accelerations during standing up and sitting down, and explore associations among trunk orientation measures across planes and transitions. Rising and lowering involve different mechanical demands, including trunk repositioning and control of the transition between sitting and standing (Sadeh et al., 2023; van der Kruk et al., 2021; Jeon et al., 2021a,b). On this basis, we formulated exploratory expectations that longer standing-up and walking durations would be associated with longer total task duration and that trunk kinematic measures would show within-transition associations across planes. These expectations are presented as exploratory rather than preregistered hypotheses. The correlation analyses were intended to describe between-participant covariance and identify candidate associations for subsequent research. They do not establish causal mechanisms or within-person temporal coordination. Phase–total correlations also involve a part–whole relationship because phase durations form part of total duration.

## 2 Materials and methods

### 2.1 Participants

Twenty-one very old women (100% female), aged 82–90 years, were recruited from community-based elderly care facilities. Their mean age was 87.95 ± 2.60 years, mean height was 153.24 ± 6.06 cm, and mean body mass was 55.33 ± 10.53 kg. All participants had both hypertension and diabetes mellitus (100% for each condition). All participants could walk independently and complete the standing-up, walking, turning, and sitting-down components of the task without assistive devices. Participants were not recruited on the basis of a specific neurological, musculoskeletal, or cognitive disorder.

#### 2.1.1 Recruitment procedure

Participants were recruited from an older adult residential facility in Fujian Province, China, in 2025. Nurses at the facility identified potential participants and invited them to participate. Thirty individuals were screened for eligibility based on their ability to walk independently without assistive devices and to independently complete all assessment procedures. Of those screened, 21 met the eligibility criteria and participated in the study.

Participants were older women who could walk independently without assistive devices and independently complete all components of the assessment task, including standing up, walking, turning, and sitting down.

#### 2.1.2 Exclusion criteria

Participants were excluded if physical or mobility impairments necessitated the use of a walking aid or other assistive device, or prevented them from independently completing the standing-up, walking, turning, or sitting-down components of the assessment.

No *a priori* sample size calculation was performed. The sample size was determined by the number of eligible participants available at the residential facility during the recruitment period in 2025. Of the 30 individuals screened, 21 met the eligibility criteria and participated in the study. This exploratory study therefore included all eligible participants identified through this recruitment process.

The study protocol was reviewed and approved by the Institutional Review Board of the local university. All experimental procedures were conducted in accordance with the ethical principles outlined in the Declaration of Helsinki and its subsequent amendments. Written informed consent was obtained from all participants and, when appropriate, their legal guardians prior to participation.

### 2.2 Experimental procedure

The experimental task followed the timed up and go (TUG) protocol, comprising a continuous sequence of standing up from a chair, walking 3 m, turning around, walking back to the chair, and sitting down (Tan et al., 2023; Maiora et al., 2024; Kataoka et al., 2023; Oliveira-Zmuda et al., 2022; Kraus et al., 2023) (Figure 1). All participants used the same armless chair, with a seat height of 45 cm, a seat width of 50 cm, and a seat depth of 50 cm. Before testing, participants sat with their feet resting naturally on the floor and their hands resting comfortably on their thighs. The IMU was secured to the posterior trunk at the level of the fifth lumbar vertebra (L5) using an elastic fixation belt and aligned with the body midline to capture trunk motion during the task. Sensor positioning and belt tightness were checked before data collection to minimize movement artifacts.

**Figure 1:**
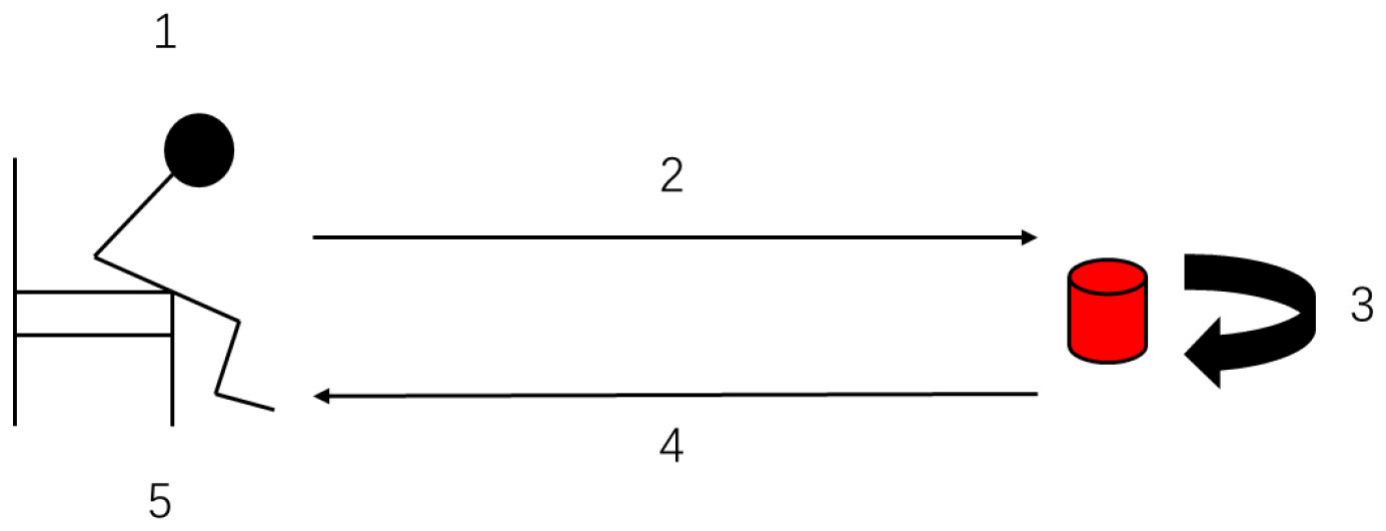
Timed up and go (TUG) functional task sequence. (1) rising from a seated position; (2) walking 3 m; (3) turning; (4) returning; and (5) sitting down again.

Upon the verbal command “start,” participants performed the entire task at a self-selected comfortable speed until they returned to a stable seated position. Kinematic data were collected using a wearable inertial measurement system (baiobit; Rivelo S.r.l., Vigonza, Italy). The sensor measured 70 × 40 × 18 mm and weighed approximately 37 g, including the battery. It incorporated triaxial accelerometers, gyroscopes, and magnetometers. The IMU was secured to the posterior trunk at L5 to measure linear accelerations in the vertical, anteroposterior, and mediolateral directions and estimate trunk orientation in the sagittal, frontal, and transverse planes, corresponding to flexion–extension, lateral bending, and axial rotation, respectively (Wang et al., 2024; De Blasiis et al., 2025; Ciotta et al., 2025). All signals were recorded and stored for subsequent processing and analysis. Each participant completed one recorded trial to reduce fatigue and testing burden.

### 2.3 Definition of task phase division

The continuous TUG sequence was segmented into six phases using predefined event-based criteria based on the task structure and IMU time-series characteristics. The standing-up phase was defined from the initiation of trunk forward flexion and seat-off to the achievement of stable upright standing. The walking-forward phase was defined from stable standing to the initiation of the first turning movement. The mid-turn phase corresponded to the primary turning movement at the 3-m mark. The walking-after-turn phase was defined from completion of the mid-turn to walking back toward the chair. The final-turn phase was defined as the turning movement performed in front of the chair to align the body for sitting. The sitting-down phase was defined from the initiation of hip and knee flexion during descent to buttock contact with the chair and achievement of a stable seated posture. Phase boundaries were identified according to these event definitions and were visually checked by the researchers to ensure consistency with the observed movement sequence:

- Standing-up phase (Stand): from the initiation of trunk forward flexion and seat-off to the achievement of a stable upright standing posture;
- Walking-forward phase (Walk forward): from stable standing to the initiation of the first turning movement;
- Mid-turn phase (Mid-turn): the primary turning movement performed at the 3 m mark;
- Walking-after-turn phase (Walk after turn): from completion of the mid-turn to walking back toward the chair;
- Final-turn phase (Final turn): the final turning movement performed in front of the chair to align the body for sitting;
- Sitting-down phase (Sit down): from the initiation of hip and knee flexion during descent to full contact of the buttocks with the chair and achievement of a stable seated position (Chow et al., 2019; Tan et al., 2023; Maiora et al., 2024; Kataoka et al., 2023; Oliveira-Zmuda et al., 2022; Kraus et al., 2023).

### 2.4 Outcome measures

Temporal outcomes comprised overall completion time and the duration of each of the six task phases, expressed in s. Phase durations were derived from their respective start and end events, as described above. Overall completion time was calculated as the sum of the segmented phase durations.

Acceleration outcomes comprised peak vertical, anteroposterior, and mediolateral trunk accelerations during standing up and sitting down, expressed in m/s^2^. The corresponding peak values were extracted from the acceleration signals within each transition.

Orientation outcomes comprised sagittal-plane flexion–extension, frontal-plane lateral bending, and transverse-plane axial rotation measures during standing up and sitting down, expressed in degrees. The maximum and minimum values were extracted separately for each transition (Moreira et al., 2025a; Zhou et al., 2025; Böttinger et al., 2024, 2025; Bochicchio et al., 2023; Wairagkar et al., 2022; Zhu et al., 2024; Chimsuwan et al., 2024; Yu and Dev, 2026; Li et al., 2023).

### 2.5 Statistical analysis

All data are presented as mean ± standard deviation (SD). The normality of continuous variables was assessed using the Shapiro–Wilk test. Pearson's product–moment correlation analysis was used to examine the relationships between overall task completion time and the duration of each segmented phase, as well as the associations among acceleration parameters, trunk orientation angle parameters, and their endpoint values, including maximum and minimum values, across different task phases.

To reduce the risk of Type I error arising from multiple comparisons, Bonferroni correction was applied within each family of correlation analyses. Both unadjusted and Bonferroni-adjusted results were considered. Correlations that were statistically significant before correction but did not remain significant after Bonferroni correction were interpreted cautiously as exploratory findings. The level of statistical significance was set at α = 0.05.

Although regression modeling may provide additional insight, a full multivariable regression model was not applied in the present study because of the small sample size and the part–whole relationship between total completion time and segmented phase durations. Specifically, total completion time was derived from the sum of the segmented phase durations; therefore, including all phase durations as predictors of total time may lead to tautological interpretation and unstable model estimates.

## 3 Results

The results showed that the total completion time for the continuous task consisting of rising from a seated position, walking 3 m, turning, returning, and sitting down was 28.20 ± 12.22 s. The durations of each segmented phase were as follows: standing up, 2.62 ± 2.86 s; walking forward, 7.31 ± 5.99 s; mid-turn, 3.40 ± 2.63 s; walking after turn, 10.36 ± 6.41 s; final turn, 2.16 ± 0.79 s; and sitting down, 2.36 ± 3.19 s (Table 1).

**Table 1:** Descriptive statistics of the overall task and segmented phase durations (*n* = 21).

Task phase	Time (s, Mean ±SD)
Overall completion time	28.20 ± 12.22
Standing up	2.62 ± 2.86
Walking forward	7.31 ± 5.99
Mid-turn	3.40 ± 2.63
Walking after turn	10.36 ± 6.41
Final turn	2.16 ± 0.79
Sitting down	2.36 ± 3.19

Values were presented as mean ± standard deviation (SD).

Overall completion time was positively correlated with walking-after-turn duration (*r* = 0.782), standing-up duration (*r* = 0.743), and walking-forward duration (*r* = 0.519). Corresponding coefficients were 0.386 for the mid-turn, 0.295 for the final turn, and 0.227 for sitting down. Standing-up duration was positively correlated with walking-after-turn duration (*r* = 0.580) and final-turn duration (*r* = 0.416; Table 2).

**Table 2:** Correlation analysis between overall task completion time and segmented phase durations (correlation coefficients).

Temporal variable	Overall completion time	Standing up	Sitting down	Walking forward	Final turn	Mid-turn	Walking after turn
Overall completion time	-						
Standing up	0.743^**^	-					
Sitting down	0.227	−0.057	-				
Walking forward	0.519^*^	0.272	−0.298	-			
Final turn	0.295	0.416^*^	0.066	−0.087	-		
Mid-turn	0.386	0.276	−0.196	0.252	−0.227	-	
Walking after turn	0.782^**^	0.580^**^	0.311	−0.010	0.395	0.092	-

Values represent Pearson correlation coefficients (r). ^**^p < 0.05. ^*^p < 0.01.

The results showed that during the overall task sequence of rising from a seated position, walking 3 m, turning, returning, and sitting down, the peak trunk accelerations during the standing-up phase were 1.11 ± 1.25 m/s^2^ in the vertical direction, 0.88 ± 1.07 m/s^2^ in the anteroposterior direction, and 0.38 ± 0.47 m/s^2^ in the mediolateral direction. During the sitting-down phase, the peak trunk accelerations were 1.12 ± 0.93 m/s^2^ in the vertical direction, 3.74 ± 1.72 m/s^2^ in the anteroposterior direction, and 1.77 ± 1.19 m/s^2^ in the mediolateral direction (Table 3).

**Table 3:** Descriptive statistics of trunk tri-axial accelerations during the standing-up and sitting-down phases (*n* = 21).

Phase	Vertical acceleration (m/s^2^)	Anteroposterior acceleration (m/s^2^)	Mediolateral acceleration (m/s^2^)
Standing up	1.11 ± 1.25	0.88 ± 1.07	0.38 ± 0.47
Sitting down	1.12 ± 0.93	3.74 ± 1.72	1.77 ± 1.19

Values are presented as mean ± standard deviation (SD).

During standing up, vertical and mediolateral peak accelerations were positively correlated (*r* = 0.561). Mediolateral peak accelerations during standing up and sitting down were also positively correlated (*r* = 0.461). The remaining coefficients ranged from −0.297 to 0.412 (Table 4).

**Table 4:** Correlation analysis of acceleration variables during the standing-up and sitting-down phases (correlation coefficients).

Acceleration variable	Standing-up vertical acceleration	Sitting-down vertical acceleration	Standing-up anteroposterior acceleration	Sitting-down anteroposterior acceleration	Standing-up mediolateral acceleration	Sitting-down mediolateral acceleration
Standing-up vertical acceleration	-					
Sitting-down vertical acceleration	−0.080	-				
Standing-up anteroposterior acceleration	0.412	−0.037	-			
Sitting-down anteroposterior acceleration	−0.123	0.266	0.007	-		
Standing-up mediolateral acceleration	0.561^**^	−0.041	−0.153	0.047	-	
Sitting-down mediolateral acceleration	0.194	−0.280	−0.190	−0.297	0.461^*^	-

Values represent Pearson correlation coefficients (r). ^**^p < 0.05. ^*^p < 0.01.

During the standing-up phase, the sagittal-plane flexion–extension angle was 27.43 ± 9.18°, the frontal-plane lateral bending angle was 4.61 ± 3.01°, and the transverse-plane axial rotation angle was 5.52 ± 3.40°. During the sitting-down phase, the sagittal-plane flexion–extension angle was 32.30 ± 16.66°, the frontal-plane lateral bending angle was 7.74 ± 9.89°, and the transverse-plane axial rotation angle was 24.43 ± 47.37° (Table 5).

**Table 5:** Descriptive statistics of trunk orientation angles during the standing-up and sitting-down phases (*n* = 21).

Phase	Sagittal plane angle/Flexion–extension (°)	Frontal plane angle/Lateral bending (°)	Transverse plane angle/Axial rotation (°)
Standing up	27.43 ± 9.18	4.61 ± 3.01	5.52 ± 3.40
Sitting down	32.30 ± 16.66	7.74 ± 9.89	24.43 ± 47.37

Values are presented as mean ± standard deviation (SD). Frontal plane angle = mediolateral (lateral bending); sagittal plane angle = flexion–extension; transverse plane angle = axial rotation.

During standing up, correlations between frontal and sagittal, frontal and transverse, and sagittal and transverse angle measures were 0.544, 0.509, and 0.603, respectively. During sitting down, the corresponding coefficients were 0.611, 0.573, and 0.951. Cross-phase coefficients ranged from −0.266 to −0.002 (Table 6).

**Table 6:** Correlation analysis of trunk orientation angles during the standing-up and sitting-down phases (correlation coefficients).

Trunk orientation angle	Standing-up frontal	Standing-up sagittal	Standing-up transverse	Sitting-down sagittal	Sitting-down frontal	Sitting-down transverse
Standing-up frontal	-					
Standing-up sagittal	0.544^**^	-				
Standing-up transverse	0.509^*^	0.603^**^	-			
Sitting-down frontal	−0.002	−0.266	−0.226	-		
Sitting-down sagittal	−0.180	−0.222	−0.170	0.611^**^	-	
Sitting-down transverse	−0.231	−0.169	−0.189	0.573^**^	0.951^**^	-

Values represent Pearson correlation coefficients (r). ^**^p < 0.05. ^*^p < 0.01.

The results showed that during the overall task sequence of rising from a seated position, walking 3 m, turning, returning, and sitting down, the maximum and minimum trunk orientation angles in flexion–extension, lateral bending, and axial rotation exhibited varying magnitudes of change across phases. During the standing-up phase, the maximum flexion–extension angle was 28.35 ± 11.00°, and the minimum flexion–extension angle was 4.09 ± 3.81°. The maximum lateral bending angle was 3.04 ± 2.58°, and the minimum lateral bending angle was 2.04 ± 1.94°. The maximum axial rotation angle was 2.96 ± 3.20°, and the minimum axial rotation angle was 3.83 ± 4.11°. During the sitting-down phase, the maximum flexion–extension angle was 34.04 ± 13.35°, and the minimum flexion-extension angle was 8.26 ± 7.01°. The maximum lateral bending angle was 6.17 ± 5.74°, and the minimum lateral bending angle was 3.83 ± 4.70°. The maximum axial rotation angle was 4.52 ± 7.94°, and the minimum axial rotation angle was 19.91 ± 48.67° (Table 7).

**Table 7:** Descriptive statistics of the maximum and minimum trunk flexion–extension, lateral bending, and axial rotation angles during the standing-up and sitting-down phases (*n* = 21).

Phase	Maximum flexion–extension angle (°)	Minimum flexion–extension angle (°)	Maximum lateral bending angle (°)	Minimum lateral bending angle (°)	Maximum axial rotation angle (°)	Minimum axial rotation angle (°)
Standing up	28.35 ± 11.00	4.09 ± 3.81	3.04 ± 2.58	2.04 ± 1.94	2.96 ± 3.20	3.83 ± 4.11
Sitting down	34.04 ±13.35	8.26 ± 7.01	6.17 ± 5.74	3.83 ± 4.70	4.52 ± 7.94	19.91 ± 48.67

Values are presented as mean ± standard deviation (SD). Flexion–extension angles correspond to sagittal-plane motion, lateral bending angles correspond to frontal-plane motion, and axial rotation angles correspond to transverse-plane motion.

Maximum and minimum flexion–extension endpoint measures were negatively correlated during standing up (*r* = −0.545) and positively correlated during sitting down (*r* = 0.419; (Table 8).

**Table 8:** Correlation analysis of joint angle endpoints (flexion–extension, lateral bending, and axial rotation) during the standing-up and sitting-down phases (correlation coefficients).

Trunk angle endpoint	Stand max FE	Sit max FE	Stand min FE	Sit min FE	Stand max LB	Sit max LB	Stand min LB	Sit min LB	Stand max Rot	Sit max Rot	Stand min Rot	Sit min Rot
Stand max FE	-											
Sit max FE	−0.024	-										
Stand min FE	−0.545^**^	0.197	-									
Sit min FE	−0.293	0.419^*^	0.341	-								
Stand max LB	0.258	−0.452^*^	−0.180	−0.274	-							
Sit max LB	−0.475^*^	0.403	0.792^**^	0.477^*^	−0.261	-						
Stand min LB	0.393	0.291	−0.086	0.510^*^	−0.200	0.048	-					
Sit min LB	−0.600^**^	0.260	0.780^**^	0.519^*^	−0.299	0.733^**^	0.051	-				
Stand max Rot	0.152	0.066	−0.033	−0.139	−0.247	0.075	−0.007	−0.049	-			
Sit max Rot	0.096	0.431^*^	−0.170	0.157	−0.324	0.240	0.211	−0.051	0.531^**^	-		
Stand min Rot	0.230	0.095	0.016	0.201	0.412	−0.159	0.349	−0.157	−0.360	−0.220	-	
Sit min Rot	−0.485^*^	0.257	0.943^**^	0.436^*^	−0.160	0.746^**^	0.049	0.843^**^	−0.137	−0.244	0.091	-

Values represent Pearson correlation coefficients (r). ^**^p < 0.05. ^*^p < 0.01. FE = flexion–extension (sagittal plane); LB = lateral bending (frontal plane); Rot = axial rotation (transverse plane).

The minimum axial rotation measure during sitting down was positively correlated with the minimum flexion–extension measure during standing up (*r* = 0.943).

The maximum lateral bending measure during sitting down correlated with minimum flexion–extension during standing up (*r* = 0.792) and sitting down (*r* = 0.477). The minimum lateral bending measure during sitting down correlated with minimum flexion–extension during standing up (*r* = 0.780) and sitting down (*r* = 0.519). Maximum and minimum lateral bending measures during sitting down were positively correlated (*r* = 0.733).

Maximum lateral bending during standing up was negatively correlated with maximum flexion–extension during sitting down (*r* = −0.452). Maximum and minimum lateral bending during sitting down were negatively correlated with maximum flexion–extension during standing up (*r* = −0.475 and −0.600, respectively).

Maximum axial rotation measures during standing up and sitting down were positively correlated (*r* = 0.531). Maximum axial rotation during sitting down correlated with maximum flexion–extension during sitting down (*r* = 0.431). Minimum axial rotation during sitting down correlated with maximum and minimum lateral bending during sitting down (*r* = 0.746 and 0.843, respectively; Table 8).

## 4 Discussion

This study describes phase-specific functional mobility in 21 very old adults. Standing-up and walking-after-turn durations had the largest observed correlations with overall completion time. Acceleration and orientation measures also showed within- and cross-transition associations. These findings describe between-participant covariance in this sample rather than identifying causal mechanisms or individual postural control strategies.

A relevant comparison is the iTUG study by Zarzeczny et al. (2017), involving 26 nursing-home women aged 80–93 years. They reported median total time of 21.06 s (IQR 9.25) and median sit-to-stand time of 2.18 s (IQR 0.97), compared with our means of 28.20 ± 12.22 s and 2.62 ± 2.86 s. Their mean stand-to-sit time was 2.75 ± 0.98 s, compared with 2.36 ± 3.19 s here. These descriptive differences cannot establish between-study effects: summary statistics, recruitment settings, and event definitions differ. Their acceleration ranges also cannot be equated with our peak accelerations. The comparison supports examining subtasks while emphasizing the need for harmonized definitions.

### 4.1 Significance of overall completion time and key phases

Walking-after-turn and standing-up durations had the largest observed correlations with total time (*r* = 0.782 and 0.743). Because each phase is included in the total, these are partly mathematical part–whole associations. They do not establish that either phase independently determines mobility, and the differences between correlation coefficients were not formally tested. Biomechanical research describes the demands of rising from sitting (Sadeh et al., 2023; Santos et al., 2021), but strength, joint kinetics, and muscle activation were not measured here. The present data therefore cannot attribute longer durations to a particular deficit.

Walking after the turn lasted 10.36 ± 6.41 s, compared with 7.31 ± 5.99 s for walking forward. This is a descriptive difference, not an inferentially tested phase effect. Reorientation and gait resumption could be relevant to the return phase (Nasruddin et al., 2024; Almajid et al., 2020), but phase boundaries, approach to the chair, and individual pacing may also affect duration. The present correlations cannot distinguish these explanations or demonstrate impaired re-acceleration.

### 4.2 Trunk acceleration characteristics during standing-up and sitting-down phases

The positive correlation between vertical and mediolateral peak accelerations during standing up (*r* = 0.561) indicates that participants with larger values in one direction tended to have larger values in the other. It does not show increased propulsive force, lateral displacement, or ineffective balance control. Likewise, the cross-phase mediolateral coefficient (*r* = 0.461) describes covariation without establishing a stable individual control strategy. Sit-to-walk research considers trunk motion alongside balance and lower-limb mechanics (van der Kruk et al., 2021; Miller et al., 2024a,b); those additional measurements would be needed to evaluate comparable mechanisms here.

The relatively small correlations involving other acceleration components should not be interpreted as evidence of independent control mechanisms. The small sample limits the precision of these estimates, and non-significant associations do not establish the absence of a relationship (Jeon et al., 2021a,b).

### 4.3 Multi-planar control of trunk orientation angles during standing-up and sitting-down phases

The positive within-transition angle correlations indicate between-participant covariation across planes. For example, a participant with a larger sagittal-plane measure could also have a larger frontal- or transverse-plane measure. Studies of postural transitions provide a basis for considering multiplanar trunk motion (Jeon et al., 2021a,b; Anan et al., 2017; Toda et al., 2023), but scalar correlations do not establish synchronized movement over time or neural coordination.

The sitting-down sagittal–transverse angle correlation was particularly large (*r* = 0.951), indicating a strong between-participant association. It does not establish synchronous movement over time. Chair alignment may provide a context for investigating multiplanar trunk motion (Toda et al., 2023; Lee et al., 2019), but the present data do not demonstrate compensatory strategies or sensory mechanisms.

### 4.4 Coupling relationships among trunk orientation angle endpoints

The negative association between maximum and minimum flexion–extension measures during standing up and the positive association during sitting down describe different between-participant patterns. Neither pattern alone establishes a particular movement strategy. Previous work has examined transition kinematics and functional status (Moreira et al., 2025b; Hwang et al., 2026); comparable interpretations would require directly measured functional or biomechanical outcomes in the present sample.

The associations between flexion–extension, lateral bending, and rotation endpoint measures provide candidate relationships for future study. Research on sitting-down and instrumented sit-to-stand performance supports examining these transitions in greater detail (Dubost et al., 2005; Van Lummel et al., 2016). However, the present scalar correlations do not demonstrate a balance-maintenance strategy or stable within-person coordination.

### 4.5 Clinical implications and study limitations

The phase-level descriptions may help formulate hypotheses about mobility assessment in samples of similarly advanced age. The present study did not test diagnostic accuracy, prospective falls, intervention responsiveness, or incremental validity over total TUG time. Clinical thresholds and targeted training recommendations therefore cannot be derived from these correlations.

Several limitations should be acknowledged. The sample comprised only 21 very old women aged 82–90 years recruited from a single older adult residential facility in Fujian Province, China. All participants had both hypertension and diabetes mellitus and could complete the task independently without assistive devices. Recruitment from a single facility and the requirement for independent mobility may have introduced selection bias. These sample characteristics limit the generalisability of the findings to men, younger older adults, individuals requiring mobility assistance, and those with different comorbidity profiles. Eligibility was restricted to participants able to complete the task independently without assistive devices. Only one recorded trial was obtained, preventing assessment of within-participant variability and test–retest reliability. No reliability analysis was reported. The cross-sectional design and bivariate correlations do not support causal inference or independent predictive effects. Phase–total duration correlations are affected by their part–whole relationship, and multiple comparisons increase the risk of chance associations. Future studies should include larger samples, repeated trials, reliability assessment, and longitudinal or intervention designs to evaluate the potential utility of phase-specific measures. Larger samples would also permit appropriately specified height-adjusted and multivariable analyses.

## 5 Conclusions

In this sample of 21 very old adults, standing-up and walking-after-turn durations had the largest observed correlations with total task duration. Trunk acceleration and orientation measures also exhibited between-participant associations during postural transitions. These findings are specific to the studied sample and should be regarded as exploratory. They do not establish causation, within-person coordination, fall prediction, or clinical superiority over total task time. Replication in larger independent samples with repeated measurements is needed.

## Data Availability

The original contributions presented in the study are included in the article/supplementary material, further inquiries can be directed to the corresponding author.
